# Physical Activity Levels, Eating Habits, and Well-Being Measures in Students of Healthcare Degrees in the Second Year of the COVID-19 Pandemic

**DOI:** 10.3390/healthcare11111570

**Published:** 2023-05-26

**Authors:** José Luis Maté-Muñoz, Juan Hernández-Lougedo, Jaime Ruiz-Tovar, Rafael Olivares-Llorente, Pablo García-Fernández, Irene Zapata

**Affiliations:** 1 Department of Radiology, Rehabilitation and Physiotherapy, Complutense University of Madrid, 28040 Madrid, Spain; jmate03@ucm.es; 2 Department of Physiotherapy, Faculty of Health Sciences, Camilo José Cela University, 28692 Madrid, Spain; jlougedo@ucjc.edu; 3 Department of Medicine, Alfonso X El Sabio University, 28691 Madrid, Spain; jpolorui@uax.es (J.R.-T.); izapamar@uax.es (I.Z.); 4 Department of Physical Activity and Sports Science, Alfonso X El Sabio University, 28691 Madrid, Spain; rolivllo@myuax.com

**Keywords:** physical activity, eating habits, toxic habits, stress, sleep, self-perceived health, COVID-19 pandemic, university students, community healthcare, lifestyle

## Abstract

Background: When the first cases of COVID-19 (caused by SARS-CoV-2 virus infection) were discovered, exceptional norms to fight the spread of the virus were established by applying movement restrictions (lockdown) in many countries. These unprecedented norms led to sedentary behaviours and less healthy diets which could persist for much longer after lockdown. The aim of this study was to analyse the physical activity, eating habits, self-perceived well-being, and toxic habits, as well as the perceived changes of these habits with respect to the pre-pandemic period, in a population of university students in the second year of the COVID-19 pandemic. Methods: A single-centre, cross-sectional study was conducted in a population of university students of healthcare degrees. A total of 961 students (639 (66.5%) women and 322 (33.5%) men) signed the informed consent and completed the questionnaire. The study was conducted through an anonymous survey, which was voluntarily self-completed by the students on an online platform. The questionnaire was based on the Spanish Health Survey and it was divided into six main parts: demographic and anthropometric characteristics, physical activity, eating habits, well-being measures (sleeping habits, health state, and stress), toxic habits, and perception of the influence of the COVID-19 pandemic on the variables described. Results and conclusions: The results showed that, during the second year of the pandemic, statistically significant dependence was identified for those students that showed higher levels of physical activity with greater perceived physical activity (*p* < 0.05), healthier eating habits (*p* < 0.05), and a better self-perceived health state (*p* < 0.05), with respect to the 12 months before the COVID-19 pandemic. On the other hand, there was a negative correlation between the sedentary students and greater perceived physical activity (*p* < 0.05). With regard to toxic habits and physical activity, a significant correlation was only detected between sedentary behaviour and cocaine consumption (*p* < 0.05). Analysing eating habits, it was observed that the students who smoked, consumed alcohol, and binge drank had low adherence to the Mediterranean diet (*p* < 0.05). In addition, those students with high stress levels slept less than 7 h (*p* < 0.05).

## 1. Introduction

Almost 30 years ago, physical activity was described as “the best buy” in public health [1]. Then, over 10 years ago, *The Lancet* published its first series on physical activity, raising awareness about its importance in the prevention of non-communicable diseases, and estimating that sedentariness is responsible for 6–10% of coronary heart disease, breast and colon cancer, and type-2 diabetes [2], posing a great public health problem with an important economic burden all over the world, defining it as “*the global pandemic of physical inactivity*” [3]. The economic burden of this pandemic of physical inactivity has been calculated to be up to 68 billion dollars worldwide [4]. The World Health Organisation (WHO) has recently published the guidelines of physical activity for children, adolescents, adults, and older people [5]. Among these guidelines, it is recommended that all adults should perform between 150 and 300 min of moderate physical activity, or between 75 and 150 min of vigorous physical activity, every week. Children and adolescents (5–18 years) are recommended to perform an average of 60 min/day of moderate–vigorous aerobic exercise [5]. However, although a larger number of countries have already implemented national strategies to promote physical activity, the levels of physical activity of the population have not increased [6].

When the first cases of COVID-19 (caused by SARS-CoV-2 virus infection) were discovered in the Chinese province of Wuhan at the end of December 2019, with the WHO declaring this new coronavirus as a pandemic on 11 March 2020 [7], exceptional norms to fight the spread of the virus were established. These unprecedented norms were implemented for people who showed symptoms (self-isolation) or had been in contact with people with symptoms (quarantine), and many countries applied restrictions on movement and daily activities in the entire community (mostly known as lockdown). For instance, during the lockdown in Spain, the state of alert was declared, affecting the whole of the Spanish territory, limiting the freedom of movement of people, who could only leave their homes to cover basic needs (e.g., buying groceries, and getting medical assistance and care for older people, children, dependent people, people with disabilities, and people in a vulnerable situation), work (if their job was essential and could not be performed at home), and visit the bank and insurance offices [8]. Spain went through 81 days of total lockdown, from March 14 (when the lockdown was ordered) to May 2 (when the de-escalation was initiated), terminating the state of alarm on 21 June 2020. Although these measures were considered necessary to limit the spread of the coronavirus, due to limited access to sport facilities, the change in daily routines, and the increase in time at home, they may have had a negative impact on the well-being of people (defined as the general term that encompasses the whole universe of scopes of human life, including the physical, mental, and social aspects, which constitute what could be called a “good life”) [7]. This deterioration of well-being has been justified, among other aspects, by the reduction of physical activity, the increase in sedentary behaviours [9,10,11,12], and the increase in unhealthier diets with a greater consumption of calories, less fruit and vegetables, and more snacks [9,13]. The sedentary behaviour is defined as “any behaviour of wakefulness characterised by a caloric expenditure of 1.5 metabolic equivalents (METs) or lower while seating, leaning or lying down” [5], and it has been evaluated in university students and staff before and after the closing down of campuses, with the participants reporting to remain seated for 7.8 more hours or 13.9% longer every week after the face-to-face lectures were suspended, reducing their total physical activity [14]. Other studies have also reported reductions in physical activity during the COVID-19 lockdown, in different countries, such as in populations of Asian older adults [15], and children and adolescents with disabilities in the United Kingdom [16]. This increase in sedentary behaviour during the COVID-19 lockdown was strongly associated with subjective mental well-being and with mood disorders, such as depression [17]. A different study reported a significant increase in the cases of depression (14%) in American (USA) adults during the pandemic with respect to previous years (9%) [18]. In the same vein, even in countries such as Korea, where the perceived physical health was maintained to a great extent thanks to the fact that the government allowed citizens to perform outdoor activities wearing masks and home exercise was promoted through online programmes, mental health was significantly affected [19]. Moreover, a study conducted with a Spanish population reported a decrease in physical activity and greater adherence to the Mediterranean diet during the lockdown [20]. However, the consumption of “unhealthy” products also increased, such as alcoholic beverages, pastries, snacks, and sweets [20]. In this sense, several studies have also reported an increase in the consumption of unhealthy snacks, sweets, desserts, and sugary drinks during the COVID-19 lockdown [21,22,23], which could have been partly due to the psychological stress caused by the lockdown, with a greater impact on young adults, ethnical minority groups, people with low economic income, and obese adults [23]. These changes, both in physical activity levels and in diet, could affect the metabolic health and well-being of people if maintained in the long-term. While metabolic variables can be measured simply and objectively, the difficulty of measuring an individual’s well-being must be taken into account, as it is a subjective variable where one would have to ask: How persistent are the subjective well-being responses of individuals who have similar routines from week to week? [24]. Still, a study by Krueger et al. [24] found reliability indices high enough to provide informative estimates for much of the current research being conducted on subjective well-being.

To sum up, the losses and routines acquired after the restrictive measures of the lockdown could persist for much longer [25], and such period could have had an impact on the health and well-being of the population. Thus, to determine whether the negative consequences of the COVID-19 lockdown were maintained or reduced in the medium-long term, the aim of this study was to analyse the physical activity, eating habits, self-perceived well-being, and toxic habits, as well as the perceived changes of these habits with respect to the pre-pandemic period, in a population of university students in the second year of the COVID-19 pandemic.

## 2. Materials and Methods

### 2.1. Study Design

A single-centre, cross-sectional study was conducted in a population of students of healthcare degrees from the Alfonso X El Sabio University in Madrid (Spain).

### 2.2. Participants

For this study, 961 students (age: 20.46 ± 3.8 years; body mass: 62.0 ± 16.0 kg; height: 1.69 ± 0.1 m; body mass index (BMI): 22.0 ± 3.4 kg·m^2^) were recruited by convenience non-probabilistic sampling, based on the following inclusion criteria: students over 18 years of age and registered in the academic year 2021–2022 in healthcare degrees at the participating university (nursing, medicine, physiotherapy, biomedicine, physical activity and sports sciences, and biomedical engineering). The study was carried out through an anonymous survey, which was voluntarily self-completed by the students on an online platform. The researchers notified participants of the confidentiality of the data to be collected and the study objectives of the research. All participants signed their consents before participating and could leave the study at any time. In addition, they did not receive any compensation for participating. Data collection was between November and December 2021. The study was authorised by the Clinical Research Bioethics Committee of the Alfonso X El Sabio University (Resolution 2021_10/099), based on the recommendations of the Declaration of Helsinki [26].

### 2.3. Measures

The questionnaire was based on the Spanish Health Survey [27], as well as on several questionnaires and validated definitions [28,29,30,31,32,33], and it was divided into six main parts: demographic and anthropometric characteristics, physical activity, eating habits, well-being measures (sleeping habits, health state, and stress), toxic habits, and perception of the influence of the COVID-19 pandemic on the variables described.

### 2.4. Demographic and Anthropometric Characteristics

The survey included age, sex, weight, height, degree, academic year when the questionnaire was completed, and different socioeconomic characteristics (economic support for studies and cohabitation). With the reported weight and height, the body mass index (BMI) (kg/m^2^) was calculated for each student. A total of 961 students (639 (66.5%) women; age: 20.44 ± 4.0 years; body mass: 56.66 ± 13.0 kg; height: 1.64 ± 0.1 m; BMI: 21.53 ± 3.1 kg·m^2^ and 322 (33.5%) men; age: 20.49 ± 3.3 years; body mass: 72.7 ± 15.9 kg; height: 1.78 ± 0.2 m; BMI: 22.87 ± 3.7 kg·m^2^) signed the informed consents and completed the questionnaires. 

### 2.5. Physical Activity

The participants were given the short version of the International Physical Activity Questionnaire (IPAQ), which consists of seven questions about physical activity performed in daily life in the last seven days, providing information about the time spent walking, conducting moderate–vigorous activities, and doing sedentary activities. Weekly physical activity was measured by recording the METs/minute/week. The reference MET values [34] are: 3.3 METs for walking, 4 METs for moderate physical activity, and 8 METs for vigorous physical activity. Based on the responses, the physical activity level of each participant was obtained, whose value corresponds to the product of the intensity (in METs) times the duration and frequency of the activity (3.3 METs/4 METs/8 METs × minutes of physical activity × days per week), classifying the subjects into three categories: (1) low (no physical activity recorded, or the physical activity recorded does not reach the medium or high categories); (2) medium (five or more days of any combination of mild, moderate, or vigorous physical activity with 600 METs/minute/week; and (3) high (three or more days of vigorous physical activity, with 1500 METs/minute/week, or seven days of any combination of mild, moderate, or vigorous physical activity, with 3000 METs/minute/week). The reliability analysis of this short version of IPAQ showed high correlation coefficients (*r* = 0.76, CI 95%: 0.73–0.77), and the concurrent validity observed between the short and long versions of IPAQ was moderately high (*r* = 0.67, CI, 95%: 0.64–0.70) [35]. Other studies have reported moderate to moderately high validity (*r* = 0.40, *p* < 0.05) [36], (*r* = 0.61, *p* < 0.05) [37], and (*r* = 0.67, *p* < 0.05) [38], with acceptable (*r* = 0.40) [39] and high reliability levels [38].

### 2.6. Eating Habits

The KIDMED questionnaire was used [40] to collect the eating habits of the participants. This questionnaire reflects an eating pattern that has been traditionally observed in Mediterranean countries and which has led to the concept of “Mediterranean diet”, characterised by a high consumption of vegetables, fruits, nuts, legumes, and cereals, a moderate consumption of fish, eggs, and dairy products, and a low consumption of meat and animal fat [41,42,43]. This questionnaire consists of 16 dichotomous questions (yes/no). Affirmative answers in the questions that represent a negative connotation regarding the Mediterranean diet (4 questions) are worth -1 point each, and affirmative answers in the questions that represent a positive aspect regarding the Mediterranean diet (12 questions) are worth +1 point each. Negative answers receive 0 points. Therefore, the said index may range between 0 (minimum adherence) and 12 points (maximum adherence). The sum of the values of the questionnaire generates the KIDMED index, which is classified into three categories: (1) from 8 to 12, optimal Mediterranean diet (high adherence); (2) from 4 to 7, must improve the eating pattern to adjust it to the Mediterranean model (medium adherence); (3) from 0 to 3, very low-quality diet (low adherence). The KIDMED questionnaire has presented plausible psychometric results, with high internal consistency (α = 0.79, CI 95 %: 0.71–0.77) and a moderate–high degree of reproducibility for each of the questions and for the entire questionnaire (κ = 0.66, CI 95 %: 0.45–0.77) [44]. In another study, similar data were reported, with moderate–excellent consistency (κ = 0.504–0.849 *p* < 0.001) in the total sample, moderate–excellent (κ = 0.497–0.803) in men, and moderate–excellent (κ = 0.435–0.927) in women [45].

### 2.7. Well-Being Measures

*Sleep duration*: The average number of hours of sleep was evaluated, considering sleep to be insufficient for values below 7 h [46,47].

*Self-rated health status*: The variable self-rated health status was used as a general indicator of health [31]. This variable was recorded through a single item. However, it has been related in numerous studies to mortality and components of subjective health appraisal [31,48]. This variable was extracted through the question “In the last 12 months, how do you consider your overall health status?”, with five possible answers; “very poor” “poor”, “fair”, “good”, and “very good”. Based on previous studies [49], participants were classified into two groups for descriptive analysis: one group of students answered “very poor”, “poor”, or “fair”, and those in the other group answered “very good” or “good”.

*Perceived stress*: Perceived stress was assessed using the perceived stress scale-10 (PSS-10) [28], validated by Remor [50] for the European population. This self-reported instrument measures the level of stress perceived during the last month by means of 10 Likert-type items. The maximum score achieved was 40 points. Following related studies, participants were classified into three levels of stress for comparison between groups: high (27–40 points); moderate (14–26 points); and low (0–13 points) [51,52]. Cronbach’s alpha was utilised to evaluate the internal consistency of the PSS-10 for the complete scale, and for the controlled item-total correlations. Based on the results obtained (α = 0.85 (0.82 to 0.87)), with mean item-total correlations ≥ 0.635, no items were eliminated as they all contributed sub-statistically to the scale.

### 2.8. Toxic Habits

*Alcohol consumption*: This variable was determined as a function of the frequency with which the participants consumed alcohol, classifying them as regular drinkers if they consumed alcoholic beverages at least once per week, and occasional drinkers if they consumed alcoholic beverages less than once per week. They were also asked whether they drank at the weekends and/or during the week. Excessive consumption of alcohol (binge drinking) was considered if the participants consumed at least 60 g of ethanol in the same day, following the definition used in the Eurostat survey [33].

*Drug use*: This was quantified through the answer to the following question: “How frequently do you consume cocaine or cannabis?” The possible answers were “never”, “occasionally”, “weekly”, or “daily” for each of the two time periods (“throughout life” and “in the last 30 days”), based on the answers reported in other studies [49].

*Smoking*: Non-smokers, active smokers, and former smokers were defined, with the latter group, including those participants who had abstained from smoking at least for the last six months. To establish the risk quotients, the sample was grouped into smokers and non-smokers.

### 2.9. Perception of the Influence of the COVID-19 Pandemic on the Variables Reported

Based on studies performed at the beginning of the pandemic [53], students were questioned about their perception of changes in measures of well-being and physical activity levels evaluated through questions such as “Comparing your current state of health with the one you had 12 months before the COVID-19 pandemic, would you say that…”, with possible responses such as: “my state of health is now better”, “my state of health is now worse”, and “my state of health is not the same”. Regarding eating and sleeping habits, participants were subjectively questioned about the possible changes in these habits. Finally, they were asked whether they had been infected with COVID-19 before the study.

### 2.10. Statistical Analysis

To compare the qualitative variables, the chi-squared test was used. The categorical variables were presented as frequencies (*n*) and percentages (%). The significance level was set at *p* < 0.05. All statistical tests were performed using the statistical package SPSS version 25.0 (SPSS, Chicago, IL, USA).

## 3. Results

### 3.1. Academic Characteristics

Of the total sample, 839 (87.3%) were Spanish students and 122 (12.7%) were foreign students. The rest of the descriptive data related to the academic characteristics of the sample are shown in Table 1.

### 3.2. Association of Students’ Perception toward the Influence of the COVID-19 Pandemic on their Health Behaviours with Physical Activity Levels

In the dependence analysis of the students’ perception toward the changes in their current physical activity levels with respect to their pre-pandemic levels (12 months before the lockdown), a statistically significant interaction was revealed (*p* < 0.05), obtaining an attraction between those students who spent more time performing physical activity (high levels) and those who perceived that they currently exercised more than before the pandemic. On the other hand, there was a negative correlation between the sedentary behaviour of the students and the perception of performing more physical activity (Table 2). The dependence analysis also revealed a significant interaction (*p* < 0.05) for greater physical activity with healthier eating habits, but not with unhealthier eating habits. Moreover, for the students’ perception toward their health state with respect to the pre-pandemic period, along with the current exercise levels, the dependence analysis showed a significant interaction (*p* < 0.05), finding an attraction between the students with high levels of physical activity and the perception of a better health state. On the other hand, there was a negative correlation between high levels of physical activity and a worse perception of the health state (Table 2). Regarding the relationship between physical activity levels, stress levels, and sleep habits, there was no significant association (*p* > 0.05).

Of all these students, 662 (68.9%) reported that they had not been infected by SARS-CoV-2, whereas 299 (31.1%) did suffer from COVID-19. Table 3 describes the participants who suffered from COVID-19 by physical activity level.

### 3.3. Associations between Healthy Behaviours and Physical Activity Levels

The dependence analysis between the perceived stress levels and physical activity levels revealed a statistically significant interaction (*p* < 0.05), observing an attraction between low stress levels and higher physical activity levels, and between higher stress levels and sedentary behaviour (Table 4).

Regarding eating habits and physical activity levels, a significant dependence was observed between these two variables (*p* < 0.05), showing a positive attraction between high adherence to the Mediterranean diet and higher physical activity levels, and between low adherence to the Mediterranean diet and sedentary behaviour. A significant dependence was also obtained between the self-perceived health state and physical activity levels (*p* < 0.05), identifying an attraction between a good self-perceived health state and high levels of physical activity, and a negative correlation was observed between a good self-perceived health state and a sedentary behaviour. Lastly, a significant interaction was detected between BMI and physical activity levels (*p* < 0.05), observing a negative correlation between higher levels of physical activity and normal weight, and a positive correlation between higher levels of physical activity and overweight, and between a sedentary behaviour and obesity. However, between sleep duration and physical activity levels, no significant interaction was revealed (*p* > 0.05).

### 3.4. Associations between Toxic Habits and Physical Activity Levels

The dependence analysis between toxic habits and physical activity levels only revealed one statistically significant interaction (*p* < 0.05) between the variable “cocaine users” and physical activity levels, observing an attraction between those individuals who used cocaine and a sedentary behavior (Table 5). For the rest of the variables related to alcohol consumption, smoking, and cannabis consumption, significant interactions were not revealed (*p* > 0.05) with respect to the physical activity levels.

### 3.5. Associations of Eating Habits with Toxic Habits and Well-Being Measures

The dependence analysis of alcohol-consumption habits and eating habits revealed a statistically significant interaction (*p* < 0.05) for the variables “drinkers”, “weekend drinkers”, and “binge drinkers” with eating habits, observing a significant attraction between all types of alcohol-users and low adherence to the Mediterranean diet (Table 6). Regarding the rest of the toxic habits, an interaction was only found for smoking, with smokers being significantly associated with worse adherence to the Mediterranean diet (*p* < 0.05). With regard to the relationship found between the variables “well-being” and “adherence to the Mediterranean diet”, significant differences were observed for sleep duration and self-perceived health state with eating habits, finding an attraction between those students who slept seven or more hours and high adherence to the Mediterranean diet, and between those students who slept less than seven hours and low adherence to the Mediterranean diet. Moreover, an attraction was found between those students who perceived their health state as “good or very good” and high adherence to the Mediterranean diet. On the other hand, a negative correlation was found between those students who perceived their health state as “fair, bad, or very bad” and high adherence to the Mediterranean diet. With respect to the relationship between adherence to the Mediterranean diet and self-perceived stress, no significant association was found (*p* > 0.05). Lastly, there was a significant correlation between high stress levels and shorter sleep duration (*p* < 0.004), whereas those students who slept more than seven hours showed lower stress levels.

Furthermore, the dependence analysis for sleep hours with self-perceived stress, smoking, and self-perceived health state showed significant differences (*p* = 0.004, *p* = 0.003, *p* = 0.030, respectively), with a minimum of seven hours of sleep being associated with low stress levels, non-smoking, and a “good or very good” self-perceived health state. On the other hand, those students who slept less than seven hours presented greater stress levels, higher smoking indices, and a “fair, bad, or very bad” self-perceived health state.

## 4. Discussion

The present study analysed the perception towards the possible changes of healthy habits before the COVID-19 pandemic (12 months before) and during the second year of the pandemic regarding the levels of physical activity. A statistically significant dependence was identified for those students who showed higher levels of physical activity with greater perceived physical activity (*p* < 0.05), healthier eating habits (*p* < 0.05), and a better self-perceived health state (*p* < 0.05), with respect to the 12 months before the COVID-19 pandemic.

On the other hand, there was a negative correlation between the sedentary students and greater perceived physical activity. These sedentary behaviours were more pronounced due to the lockdown measures to limit the spread of the SARS-CoV-2 coronavirus [10,11,12]. Moreover, a study conducted after the second lockdown in May 2021 in a large sample of physicians in Saudi Arabia showed a low physical activity, whereas the prevalence of musculoskeletal disorders was high [54]. However, in our study, those students who performed more physical activity had a healthier lifestyle during the second year of the COVID-19 pandemic. In this sense, the study of Lombardo et al. [55] suggested that physical activity, especially in the morning, increased considerably during the COVID-19 pandemic with respect to the pre-lockdown period in March 2020. Furthermore, the longer time that the population spent at home allowed them to be more flexible to prepare fresh meals, and prevented them from spending time in bars and pubs [55]. Therefore, it seems that those who spend more time doing sports improve their healthy habits even in a pandemic situation. Regarding the above-mentioned, our study established significant associations for those students who spent more time performing physical activity with low stress levels, greater adherence to the Mediterranean diet, and a better self-perceived health state. This is especially important, due to the negative effects that the COVID-19 disease can have on people, at the CNS and endothelial levels, and this response can vary in patients with different levels of physical activity. This was analysed by Freire et al. [56], who reported that the physically inactive individuals that had been infected by SARS-CoV-2 presented a significantly greater sympathetic activity and a lower parasympathetic activity than the active individuals of the control group who had not been infected [56]. Thus, it would be interesting to design and apply new strategies aimed at overcoming the negative effects of the pandemic and at implementing preventive measures to facilitate the recovery of the current cases of COVID-19. One of such measures would include the improvement of sleep hygiene. Regarding prevention, sleep participates in the regulation and functioning of the immune system [57]. Another measure to improve the respiratory system along with sleep quality is physical exercise, which has been widely cited in the literature [58]. In the present study, no significant differences were observed for the different physical activity levels with sleep hygiene or duration. However, in a recent study, the state of well-being could have been affected by sleep quality (being bad in 51% of women and in 47% of men) and by sleep duration (17.4% of women and 19.7% of men slept less than 5 h) two years after the COVID-19 pandemic [59]. It would, therefore, be interesting for health professionals to implement strategies to increase both the duration and quality of sleep.

With regard to toxic habits and physical activity, a significant correlation was only detected between sedentary behaviour and cocaine consumption. Therefore, a sedentary behaviour could lead to toxic habits that worsen health and may increase the secondary effects derived from a possible infection by SARS-CoV-2. A recent study of Zapata et al. [60] analysed the toxic habits (especially smoking and alcohol consumption) of university students, relating them to insufficient sleep duration and a worse self-perceived health state during the second year of the COVID-19 pandemic, finding no correlation with high stress levels [60]. Risk drinkers (33.2%) were associated with being female, and the consumption of cannabinoids (6.7%) was associated with being male [60]. Nevertheless, other studies conducted in university populations did find an association for smoking and alcohol consumption with higher stress levels [53,61,62]. Therefore, it seems necessary to take measures to reduce toxic habits and stress, exercise being one such measure.

Analysing eating habits, it was observed that the students who smoked, consumed alcohol, and binge drank had low adherence to the Mediterranean diet. Moreover, there was a correlation for those students with a “good or very good” self-perceived health state with higher physical activity levels and greater adherence to the Mediterranean diet. In the same line, other studies have reported that the positive changes in eating habits observed during the months of lockdown (eating fresh foods, fruits, vegetables, dairy products, etc.) were significantly associated with higher income, a better self-perceived health state and better sport habits [63], more time available for cooking [55], and simply eating at home [64]. Another study in a sample of adolescents with chronic affectations showed changes in eating habits, with a decrease in the consumption of precooked foods and an increase in homemade food [65]. However, there was also an increase in the proportion of meals that were eaten while watching TV [65]. Furthermore, a recent study that analysed the impact perceived two years after the beginning of the COVID-19 pandemic in young adults showed that 48% of the individuals perceived that they had gained weight after the pandemic, which was associated with a self-declared increase in the number of meals per day (≥ 4), with an increase in screen time by almost 2.5 h per day [66]. Although it has not been demonstrated that anxiety and stress are strongly related to a greater consumption of unhealthy products, such as ultra-processed foods, sweets, and chocolate in university populations [67], our study could not demonstrate a significant relationship between the students with high stress levels and low adherence to the Mediterranean diet. However, it was observed that those students with high stress levels slept less than 7 h. This maintenance of the state of anxiety during the lockdown period in the students, along with the levels of insomnia, could have been due to the access to the internet through smartphones, since they received news about the evolution of the pandemic and the daily number of deaths [68]. On the other hand, a different study reported increases in the duration of sleep during the lockdown period in a population of young adults [69]. Although the COVID-19 pandemic negatively affected most sporting activities at all levels, it also opened up new positive perspectives for sport, with university students being a target group [70]. Therefore, it will be necessary to generate in these students, on the one hand, correct eating habits, such as guidelines on Mediterranean diet, and on the other hand, to implement physical activity programmes, using new technologies or developing existing ones (online communication technologies, distance coaching, training in the home environment), which help to reduce toxic habits, stress, and anxiety, and, in turn, increase sleep hygiene and their perception of health status.

### Limitations

Since this was a cross-sectional study, we could not establish causal relationships. In addition, random sampling and a follow-up of the population with a posterior survey could improve the quality of the study. Nevertheless, the students were supervised by a researcher while they completed the questionnaire (solving all the questions in the questionnaire), which could have improved the quality of the answers.

Selection bias may have occurred, since data were gathered only from the questionnaires that were completed by the students who attended the lectures; thus, we lack the data of the students who usually miss the lectures, and such absenteeism is sometimes related to the worse health habits. Furthermore, since the measurement instrument was a self-informed questionnaire, it may have underestimated the consumption of substances due to social desirability bias. Nevertheless, these instruments have been validated in university populations to analyse the consumption of substances [71].

Lastly, this study was based on the retrospective recall of the students, as they were asked about the changes perceived since the period before the COVID-19 pandemic, with the possibility of being affected by memory bias [72]. Thus, these results must be considered as individual perceptions.

Bearing in mind these limitations, further research should be related to random sampling designs at different universities, so that the results can be generalised to a wider population. It would also be interesting to include an exercise test that includes the measurement of some physiological variable.

## 5. Conclusions

During the second year after the pandemic, those students with higher levels of physical activity had a healthier lifestyle as they had a higher adherence to the Mediterranean diet and a better health perception compared to the 12 months prior to the COVID-19 pandemic. In addition, those students who smoked and consumed alcohol had poor eating habits. Therefore, it would be very important to generate lines of action for university students aimed at improving their levels of physical activity, either through supervised exercise programmes at the university itself or in municipal centres, or through university sport. In this way, young adults could acquire healthy lifestyles, reducing toxic habits and unhealthy diets.

## Figures and Tables

**Table 1:** Academic and socioeconomic characteristics among the participants 12 months before COVID-19 pandemic (*n* = 961).

Academic Major	*n* (%)
Medicine	361 (37.5%)
Nursing	350 (36.4%)
Physiotherapy	90 (9.4%)
Sport Sciences	74 (7.7%)
Biomedicine	70 (7.3%)
Biomedical Engineering	16 (1.7%)
**Year**	***n* (%)**
1	417 (43.4%)
2	337 (35.1%)
3	90 (9.4%)
4	59 (6.1%)
5	32 (3.3%)
6	26 (2.7%)
**Coexistence during the Academic Year**	***n* (%)**
In the family home	409 (42.6%)
In a shared flat	265 (27.6%)
In a student residence	223 (23.2%)
Living alone	64 (6.6%)
**Economic support for University cost**	***n* (%)**
Family	847 (88.2%)
Student’s job	58 (6%)
Grants	56 (5.8%)

PA =Physical Activity.

**Table 2:** Correlations between perceived changes in physical activity habits, eating habits, health state, stress levels, and sleep habits with the current physical activity levels.

	Physical Activity Levels					
Do you think that your physical activity habits have changed with respect to 12 months before the COVID-19 pandemic?	Sedentary (*n*)	PA Low (*n*)	PA Medium (*n*)	PA High (*n*)	Total (*n*)	*p*
It has not changed.	27 (45.8%)	51 (34.5%)	55 (28.2%)	153 (29.8%)	210 (21.8%)	<0.001 *
Yes, I currently do more physical activity.	8 (13.6%)	13 (8.8%)	40 (20.5%)	244 (43.6%)	305 (31.7%)	
Yes, I currently do less physical activity.	24 (40.7%)	84 (56.8%)	100 (51.3%)	162 (29%)	370 (38,5%)	
Total	59 (100%)	148 (100%)	195 (100%)	559 (100%)	961 (100%)	
**Do you think that your** **eating habits have** **changed with respect to** **12 months before the COVID-19 pandemic?**	**Sedentary** **(*n*)**	**PA** **Low** **(*n*)**	**PA** **Medium** **(*n*)**	**PA** **High** **(*n*)**	**Total** **(*n*)**	***p***
No, my eating habits are the same.	35 (59.3%)	71 (48%)	104 (53.3%)	292 (52.2%)	502 (15.4%)	0.023 *
Yes, my eating habits are now healthier.	27 (23.7%)	92 (27.7%)	107 (23.1%)	267 (32.4%)	493 (29.2%)	
Yes, my eating habits are now unhealthier.	14 (16.9%)	25 (24.3%)	59 (23.6%)	222 (15.4%)	320 (18.5%)	
Total	59 (100%)	148 (100%)	195 (100%)	559 (100%)	961 (100%)	
**Comparing your current health state with that** **which you had 12 months before the COVID-19** **pandemic, would you say that your current health state is better or worse?**	**Sedentary** **(*n*)**	**PA** **Low** **(*n*)**	**PA** **Medium** **(*n*)**	**PA** **High** **(*n*)**	**Total** **(*n*)**	***p***
The same.	25 (42.4%)	67 (45.3%)	88 (45.1%)	223 (39.9%)	403 (41.9%)	<0.001 *
My health status is better.	13 (22%)	29 (19.6%)	46 (23.6%)	202 (36.1%)	290 (30.2%)	
My health status is worse.	21 (35.6%)	52 (35.1%)	61 (31.3%)	134 (24%)	268 (27.9%)	
Total	59 (100%)	148 (100%)	195 (100%)	559 (100%)	961 (100%)	
**Comparing your current stress level with that which you had 12 months before the COVID-19 pandemic, would you say that** **your current stress level** **is greater or lower?**	**Sedentary** **(*n*)**	**PA** **Low** **(*n*)**	**PA** **Medium** **(*n*)**	**PA** **High** **(*n*)**	**Total** **(*n*)**	***p***
I have the same stress level now.	20 (33.9%)	39 (26.4%)	51 (26.2%)	158 (28.3%)	268 (27.9%)	0.782
My current stress level is lower.	9 (15.3%)	33 (22.3%)	47 (24.1%)	132 (23.6%)	221 (23%)	
My current stress level is higher.	30 (50.8%)	76 (51.4%)	97 (49.7%)	269 (48.1%)	472 (49.1%)	
Total	59 (100%)	148 (100%)	195 (100%)	559 (100%)	961 (100%)	
**Do you think that your sleep habit has** **changed with respect to** **12 months before the** **COVID-19 pandemic?**	**Sedentary** **(*n*)**	**PA** **Low** **(*n*)**	**PA** **Medium** **(*n*)**	**PA** **High** **(*n*)**	**Total** **(*n*)**	***p***
No, it is the same.	21 (35.6%)	61 (41.2%)	79 (40.5%)	232 (41.5%)	393 (40.9%)	0.856
Yes, it is better now.	9 (15.3%)	24 (16.2%)	33 (16.9%)	75 (13.4%)	141 (14.7%)	
Yes, it is worse now.	29 (49.2%)	63 (42.6%)	83 (42.6%)	252 (45.1%)	427 (44.4%)	
Total	59 (100%)	148 (100%)	195 (100%)	559 (100%)	961 (100%)	

PA = physical activity, * = significant differences (*p* < 0.05).

**Table 3:** SARS-CoV-2-infected participants according to different levels of current physical activity.

	Have you Suffered from COVID-19?		
Physical Activity Levels	No	Yes	Total
Sedentary	41 (6.2%)	18 (6.1%)	59 (6.1%)
Low	101 (15.3%)	47 (15.7%)	148 (15.4%)
Medium	127 (19.2%)	68 (22.7%)	195 (20.3%)
High	393 (59.3%)	166 (55.5%)	559 (58.2%)
Total	662	299	961

**Table 4:** The relationship of stress perception, eating habits, sleep duration, health status, and body mass index with the current physical activity levels.

Physical Activity Levels						
Stress perception (PSS-10)	Sedentary (*n*)	PA Low (*n*)	PA Medium *(n*)	PA High (*n*)	Total	*p*
Low	5 (8.5%)	24 (16.2%)	35 (18%)	148 (26.5%)	210 (21.8%)	0.002 *
Medium	38 (64.4%)	91 (61.5%)	120 (61.5%)	329 (58.9%)	578 (60.2%)	
High	16 (27.1%)	33 (22.3%)	40 (20.5%)	82 (14.7%)	173 (18%)	
Total	59 (100%)	148 (100%)	195 (100%)	559 (100%)	961 (100%)	
**Eating habits (KIDMED)**	**Sedentary** **(*n*)**	**PA Low** **(*n*)**	**PA Medium** ***(n*)**	**PA High** **(*n*)**	**Total**	***p***
Poor	18 (30.5%)	31 (20.9%)	29 (14.9%)	70 (12.5%)	148 (15.4%)	<0.001 *
Medium	27 (45.8%)	92 (62.2%)	107 (54.9%)	267 (47.8%)	493 (51.3%)	
High	14 (23.7%)	25 (16.9%)	59 (30.3%)	222 (39.7%)	320 (33.3%)	
Total	59 (100%)	148 (100%)	195 (100%)	559 (100%)	961 (100%)	
**Sleep duration**	**Sedentary** **(*n*)**	**PA Low** **(*n*)**	**PA Medium** ***(n*)**	**PA High** **(*n*)**	**Total**	***p***
<7 h	32 (54.2%)	61 (41.2%)	88 (45.1%)	259 (46.3%)	440 (45.8%)	0.385
≥7 h	27 (45.8%)	87 (58.8%)	107 (54.9%)	300 (53.7%)	521 (54.2%)	
Total	59 (100%)	148 (100%)	195 (100%)	559 (100%)	961 (100%)	
**Self-assessment of** **health status**	**Sedentary** **(*n*)**	**PA Low** **(*n*)**	**PA Medium** ***(n*)**	**PA High** **(*n*)**	**Total**	***p***
Good–very good	36 (61%)	92 (62.2%)	133 (68.2%)	417 (74.6%)	678 (70.6%)	0.006 *
Fair–bad–very bad	23 (39%)	56 (37.8%)	62 (31.8%)	142 (25.4%)	283 (29.4%)	
Total	59 (100%)	148 (100%)	195 (100%)	559 (100%)	961 (100%)	
**BMI**	**Sedentary** **(*n*)**	**PA Low** **(*n*)**	**PA Medium** ***(n*)**	**PA High** **(*n*)**	**Total**	***p***
Underweight	8 (13.6%)	29 (19.6%)	23 (11.8%)	45 (8.1%)	105 (10.9%)	0.015 *
Normal weight	42 (71.2%)	99 (66.9%)	146 (74.8%)	402 (71.9%)	689 (71.7%)	
Overweight	7 (11.8%)	18 (12.2%)	22 (11.3%)	103 (18.4%)	150 (15.6%)	
Obesity	2 (3.4%)	2 (1.3%)	4 (2.1%)	9 (1.6%)	17 (1.8%)	
Total	59 (100%)	148 (100%)	195 (100%)	559 (100%)	961 (100%)	

PA = physical activity, PSS-10 = perceived stress scale-10, BMI = body mass index, * = significant differences (*p* < 0.05).

**Table 5:** The relationship between toxic habits and physical activity levels.

Physical Activity Levels						
Regular drinker	Sedentary (*n*)	PA Low (*n*)	PA Medium (*n*)	PA High (*n*)	Total (*n*)	*p*
No	5 (8.5%)	9 (6.1%)	21 (10.8%)	74 (13.2%)	109 (11.3%)	0.085
Yes	54 (91.5%)	139 (93.9%)	174 (89.2%)	485 (86.8%)	852 (88.7%)	
Total	59 (100%)	148 (100%)	195 (100%)	559 (100%)	961 (100%)	
**Weekend** **drinker**	**Sedentary (*n*)**	**PA Low** **(*n*)**	**PA Medium (*n*)**	**PA High** **(*n*)**	**Total** **(*n*)**	***p***
No	15 (25.4%)	38 (25.7%)	47 (24.1%)	149 (26.7%)	249 (25.9%)	0.918
Yes	44 (74.6%)	110 (74.3%)	148 (75.9%)	410 (73.3%)	712 (74.1%)	
Total	59 (100%)	148 (100%)	195 (100%)	559 (100%)	961 (100%)	
**Weekday** **drinker**	**Sedentary (*n*)**	**PA Low** **(*n*)**	**PA Medium (*n*)**	**PA High** **(*n*)**	**Total** **(*n*)**	***p***
No	41 (69.5%)	95 (64.2%)	121 (62.1%)	368 (65.8%)	625 (65%)	0.687
Yes	18 (30.5%)	53 (35.8%)	74 (37.9%)	191 (34.2%)	336 (35%)	
Total	59 (100%)	148 (100%)	195 (100%)	559 (100%)	961 (100%)	
**Binge** **drinker**	**Sedentary (*n*)**	**PA Low** **(*n*)**	**PA Medium (*n*)**	**PA High** **(*n*)**	**Total** **(*n*)**	***p***
No or <once/month	48 (81.4%)	117 (79.1%)	159 (81.5%)	427 (76.4%)	751 (70.6%)	0.435
Yes	11 (18.6%)	31 (20.9%)	36 (18.5%)	132 (23.6%)	210 (29.4%)	
Total	59 (100%)	148 (100%)	195 (100%)	559 (100%)	961 (100%)	
**Smoker**	**Sedentary (*n*)**	**PA Low** **(*n*)**	**PA Medium (*n*)**	**PA High** **(*n*)**	**Total** **(*n*)**	***p***
No	32 (54.2%)	95 (64.2%)	135 (69.2%)	388 (69.4%)	650 (67.6%)	0.083
Yes	27 (45.8%)	53 (35.8%)	60 (30.8%)	171 (30.6%)	311 (32.4%)	
Total	59 (100%)	148 (100%)	195 (100%)	559 (100%)	961 (100%)	
**Cannabis** **smoker**	**Sedentary (*n*)**	**PA Low** **(*n*)**	**PA Medium (*n*)**	**PA High** **(*n*)**	**Total** **(*n*)**	***p***
No	46 (78%)	134 (90.5%)	167 (85.6%)	464 (83%)	811 (84.4%)	0.067
Yes	13 (22%)	14 (9.5%)	28 (14.4%)	95 (17%)	150 (15.6%)	
Total	59 (100%)	148 (100%)	195 (100%)	559 (100%)	961 (100%)	
**Cocaine** **consumer**	**Sedentary (*n*)**	**PA Low** **(*n*)**	**PA Medium (*n*)**	**PA High** **(*n*)**	**Total** **(*n*)**	***p***
No	52 (88.1%)	145 (98%)	192 (98.5%)	535 (95.7%)	924 (10.8%)	0.002 *
Yes	7 (11.9%)	3 (2%)	3 (1.5%)	24 (4.3%)	37 (73.3%)	
Total	59 (100%)	148 (100%)	195 (100%)	559 (100%)	961 (100%)	

PA = physical activity, * = significant differences (*p* < 0.05).

**Table 6:** The relationship of eating habits with toxic habits and measures of wellbeing.

Eating habits (KIDMED)						
Drinker	Low (*n*)	Medium (*n*)	High (*n*)	Total (*n*)	*p*	
No	15 (10.1%)	43 (8.7%)	50 (15.7%)	108 (11.2%)	0.006 *	
Yes	133 (89.9%)	451 (91.3%)	269 (84.3%)	853 (88.8%)		
Total	148 (100%)	494 (100%)	319 (100%)	961 (100%)		
**Weekend** **drinker**	**Low** **(*n*)**	**Medium** **(*n*)**	**High** **(*n*)**	**Total** **(*n*)**	***p***	
No	30 (20.3%)	114 (23.1%)	104 (32.6%)	248 (25.8%)	0.002 *	
Yes	118 (79.7%)	380 (76.9%)	215 (67.4%)	713 (74.2%)		
Total	148 (100%)	494 (100%)	319 (100%)	961 (100%)		
**Weekday** **drinker**	**Low** **(*n*)**	**Medium** **(*n*)**	**High** **(*n*)**	**Total** **(*n*)**	***p***	
No	88 (59.5%)	319 (64.6%)	218 (68.4%)	625 (65%)	0.157	
Yes	60 (40.5%)	175 (35.4%)	101 (31.6%)	336 (35%)		
Total	148 (100%)	494 (100%)	319 (100%)	961 (100%)		
**Binge** **drinker**	**Low** **(*n*)**	**Medium** **(*n*)**	**High** **(*n*)**	**Total** **(*n*)**	***p***	
No or < once/month	105 (70.9%)	385 (77.9%)	261 (81.8%)	751 (78.1%)	0.026 *	
Yes	43 (29.1%)	109 (22.1%)	58 (18.2%)	210 (21.9%)		
Total	148 (100%)	494 (100%)	319 (100%)	961 (100%)		
**Smoker**	**Low** **(*n*)**	**Medium** **(*n*)**	**High** **(*n*)**	**Total** **(*n*)**	***p***	
No	86 (58.1%)	327 (66.2%)	237 (74.3%)	650 (67.6%)	<0.001 *	
Yes	62 (41.9%)	167 (33.8%)	82 (25.7%)	311 (32.4%)		
Total	148 (100%)	494 (100%)	319 (100%)	961 (100%)		
**Cannabis** **smoker**	**Low** **(*n*)**	**Medium** **(*n*)**	**High** **(*n*)**	**Total** **(*n*)**	***p***	
No	127 (85.8%)	410 (83%)	274 (83%)	811 (84.4%)	0.463	
Yes	21 (14.2%)	84 (17%)	45 (17%)	150 (15.6%)		
Total	148 (100%)	494 (100%)	319 (100%)	961 (100%)		
**Cocaine** **consumer**	**Low** **(*n*)**	**Medium** **(*n*)**	**High** **(*n*)**	**Total** **(*n*)**	***p***	
No	140 (94.6%)	478 (96.8%)	306 (95.9%)	924 (96.1%)	0.471	
Yes	8 (5.4%)	16 (3.2%)	13 (4.1%)	37 (3.9%)		
Total	148 (100%)	494 (100%)v	319 (100%)	961 (100%)		
**Sleep** **duration**	**Low** **(*n*)**	**Medium** **(*n*)**	**High** **(*n*)**	**Total** **(*n*)**	***p***	
< 7 h	84 (56.8%)	230 (46.6%)	126 (39.5%)	440 (45.8%)	0.002 *	
≤ 7 h	64 (43.2%)	264 (53.4%)	193 (60.5%)	521 (54.2%)		
Total	148 (100%)	494 (100%)	319 (100%)	961 (100%)		
**Self-assessment** **of health status**	**Low** **(*n*)**	**Medium** **(*n*)**	**High** **(*n*)**	**Total** **(*n*)**	***p***	
Good—very good	83 (56.1%)	334 (67.6%)	261 (81.8%)	678 (70.6%)	<0.001 *	
Fair—bad—very bad	65 (43.9%)	160 (32.4%)	58 (18.2%)	283 (29.4%)		
Total	148 (100%)	494 (100%)	319 (100%)	961 (100%)		
**Stress perception (PSS-10)**	**Low** **(*n*)**	**Medium** **(*n*)**	**High** **(*n*)**	**Total** **(*n*)**	***p***	
Low	30 (20.3%)	98 (19.9%)	81 (25.4%)	209 (21.8%)	0.072 *	
Medium	88 (59.4%)	296 (59.9%)	194 (60.8%)	578 (60.2%)		
High	30 (20.3%)	100 (20.2%)	44 (13.8%)	174 (18%)		
Total	148 (100%)	494 (100%)	319 (100%)	961 (100%)		

PSS-10 = perceived stress scale-10, * = significant differences (*p* < 0.05).

## Data Availability

The datasets used and/or analyzed during the current study are available from the corresponding author upon reasonable request.
